# Health care resources and costs associated with delivering gene therapy for hemophilia in clinical practice

**DOI:** 10.1016/j.rpth.2025.103275

**Published:** 2025-12-02

**Authors:** Diaz M. Prameyllawati, Caroline M.A. Mussert, Martijn A.H. Oude Voshaar, Hester F. Lingsma, Marjon H. Cnossen, Michiel Coppens, Karina Meijer, Paul R. van der Valk, Frank W.G. Leebeek, Renske M.T. ten Ham

**Affiliations:** 1 Department of Public Health, Erasmus MC, University Medical Center Rotterdam, Rotterdam, the Netherlands; 2 Department of Epidemiology and Health Economics, Julius Center for Health Sciences and Primary Care, University Medical Center Utrecht, Utrecht, the Netherlands; 3 Department of Pediatric Hematology and Oncology, Erasmus MC Sophia Children’s Hospital, University Medical Center Rotterdam, Rotterdam, the Netherlands; 4 Department of Vascular Medicine, Amsterdam University Medical Center, University of Amsterdam, Amsterdam, the Netherlands; 5 Amsterdam Cardiovascular Sciences, Pulmonary Hypertension & Critical Care, Amsterdam, the Netherlands; 6 Department of Hematology, University Medical Center Groningen, University of Groningen, Groningen, the Netherlands; 7 Center for Benign Hematology, Thrombosis and Hemostasis, Van Creveldkliniek, University Medical Center Utrecht, Utrecht University, Utrecht, the Netherlands; 8 Department of Hematology, Erasmus MC, University Medical Center Rotterdam, Rotterdam, the Netherlands

**Keywords:** hemophilia, gene therapy, health care resource, cost, microcosting

## Abstract

**Background:**

The debate around the cost-effectiveness of gene therapy for hemophilia has largely centered on its price (€1-€3.5 million per individual). While previous studies have explored care organization for gene therapy delivery, none have evaluated the potential resource utilization and associated costs in a real-world setting.

**Objectives:**

This study aimed to estimate the health care resources and costs of delivering gene therapy for hemophilia in clinical practice.

**Methods:**

We conducted a bottom-up microcosting study and constructed a process map outlining each step of care. Data on resource use were obtained from clinical trial protocols and translated to reflect real-world clinical practice through semistructured interviews. Dutch unit costs were assigned to each resource, and mean total costs per individual were calculated for hemophilia A and B. Sensitivity analyses were performed to assess the potential range of consumed resources and costs.

**Results:**

In clinical practice, delivering gene therapy for hemophilia is expected to require resources such as personnel time, hospital visits, laboratory tests, liver function assessments, drugs, hospital facilities, medical consumables, and office equipment. The estimated total cost for an eligible individual without liver function abnormalities, covering screening, pretreatment preparation, administration, and first-year follow-up, is €28,696 (€20,873-€48,973) for hemophilia A and €20,511 (€18,175-€36,310) for hemophilia B.

**Conclusion:**

Delivery of hemophilia gene therapy requires significant resources, incurs substantial costs, and demands additional organizational infrastructure within treatment facilities. These findings may aid stakeholders to better plan implementation of these innovative therapies into clinical practice, as well as inform economic evaluations and reimbursement discussions.

## Essentials

• Implementing gene therapy for hemophilia in clinical practice may incur notable delivery costs.
• This microcosting study combined trial data with expert interviews to estimate delivery costs.
• Estimated delivery costs on average: €28,696 (hemophilia A) and €20,511 (hemophilia B).
• Our findings inform clinical implementation, economic evaluations, and reimbursement discussions.

## 1 Introduction

Hemophilia is a rare congenital bleeding disorder characterized by a deficiency of coagulation factor (F)VIII (hemophilia A) or FIX (hemophilia B), leading to a bleeding tendency [1,2]. The hallmark of hemophilia treatment is replacement therapy with factor concentrates or nonfactor therapies to ensure adequate hemostasis [3]. In the Netherlands, prophylaxis with factor concentrates or nonfactor therapies, in particular emicizumab for hemophilia A, constitutes the standard of care [4]. While prophylactic therapy has significantly extended both the lifespan and quality of life of individuals living with hemophilia, it remains burdensome due to the necessity of lifelong regular injections [5].

One of the recent treatment advancements for hemophilia is gene therapy (GT). GT alleviates the burden of current therapies by a single infusion that induces long-term sufficient expression of FVIII or FIX to secure hemostasis [6,7]. To date, 2 GT products have been approved in the European Union. Valoctocogene roxaparvovec (Roctavian, BioMarin) has conditional marketing authorization for hemophilia A, and etranacogene dezaparvovec (Hemgenix, CSL Behring) is approved for hemophilia B [6]. These GTs for hemophilia contain a genetically modified adeno-associated virus (AAV) delivering the FVIII or FIX gene to the liver [6]. Clinical trials indicate that these GTs achieve significant reduction of bleeding and eliminate the need for prophylaxis [8]. An increasing number of GTs for hemophilia are currently in advanced clinical trial phases and are likely to seek regulatory approval within the next few years [9].

Regulations and guidance documents dictate that GT must be administered in a controlled, specialized hospital setting at centers with established expertise in GT [[10], [11], [12], [13]]. In addition, to assess eligibility a thorough screening process is required, as well as subsequent follow-up up to 15 years after infusion [14,15]. Few (early) economic evaluations have assessed cost-effectiveness of these GTs compared with prophylaxis withFVIII/FIX or emicizumab [7,[16], [17], [18], [19], [20]]. However, the debates on cost-effectiveness predominantly focus on the pricing of these GTs themselves, which are reported to range from €1 to €3.5 million per infusion [21,22]. Several guidance documents have been published to support the implementation of hemophilia GTs in clinical practice [[10], [11], [12], [13]]. However, publications detailing the required resources and their associated costs in delivering GT on a hospital level are lacking.

GT treatment demands additional resources compared with standard hemophilia care [10]. Its implementation is expected to require lasting changes in hospital care organization, increased workload and training needs, and impacting routine clinical workflows. For successful and sustainable implementation of GTs in clinical practice, a better understanding of resource utilization and associated costs is necessary. Therefore, this study aimed to estimate the health care resources and costs of delivering GT for hemophilia in clinical practice.

## 2 Methods

### 2.1 Study design and scope

We conducted a bottom-up microcosting study, which first entailed identification of resource use through a process map. Next, the identified resources were assigned unit costs and aggregated to estimate the mean site preparation costs per hospital and the mean GT delivery costs per individual.

In most European Union countries including the Netherlands, the administration of hemophilia GTs is currently limited to clinical trials. Hence, we estimated real-world resource utilization using data derived from clinical trial protocols, supplemented by insights gathered through semistructured interviews with healthcare professionals experienced in hemophilia GTs. Data on resource use were obtained from summary product characteristics (SMPCs) and protocols of 3 multicenter clinical trials: BMN-270 (GENEr8-1; NCT03370913), AMT-060 (NCT02396342, using a wild-type FIX gene construct), and AMT-061 (HOPE-B; NCT03489291, using a Factor IX Padua gene construct). BMN-270 evaluated valoctocogene roxaparvovec [23], while AMT-061 evaluated etranacogene dezaparvovec [24,25].

For the delivery costs associated with hemophilia GTs, 3 distinct treatment scenarios were defined to represent the potential clinical pathways that individuals followed during the care delivery process. The first scenario estimated costs for individuals who were screened but noneligible for GT. The second covered individuals who were screened, found eligible, were treated and remained free of liver function abnormalities during the follow-up period. The third included individuals who were screened, found eligible, were treated but developed liver function abnormalities during the follow-up period. Additionally, estimation of second-year follow-up costs was undertaken to support long-term cost projections. The first year of follow-up was characterized by a more intensive protocol [14,15], whereas follow-up in the second year and subsequent years followed a more standardized protocol. Furthermore, sensitivity analyses were performed to estimate the minimum and maximum costs.

A hospital perspective was applied, meaning that calculated costs refer to the expenses incurred by hospitals to deliver GTs, excluding resources consumed outside the hospital [26]. In the Netherlands, centers that participated in administering hemophilia GTs during clinical trials are expected to deliver the complete care of GT in routine care, encompassing both infusion and follow-up. Accordingly, we assumed that the costs estimated in this study reflect this specific context, namely centers with prior trial experience that are anticipated to provide both infusion and follow-up in clinical practice.

Since this study specifically focused on the costs involved in delivering GT, we did not include a cost-effectiveness comparison with the standard of care. Therefore, the costs of standard care were considered beyond the scope of this analysis. Furthermore, costs associated with clinical trial execution such as ethics committee fees, trial management, and other study-related expenses, were excluded, as the analysis concentrated solely on the delivery aspects of GT rather than the resources required to initiate and conduct clinical trials.

### 2.2 Process map

The process of delivering hemophilia GTs was visualized in a chronological flowchart, referred to as a process map [27]. The development of this map comprised 2 phases. First, a preliminary process map was constructed based on trial protocols and SMPCs using Microsoft Visio (Microsoft). In the second phase, semistructured interviews were used to iteratively refine this preliminary map, which was shared with the participants for final feedback and approval. The final version of the process map presents all possible care activities throughout the GT delivery process irrespective of hemophilia type. The starting point is the site preparation phase, with the endpoint marked by the conclusion of the follow-up phase. In the process map we have made transparent which activities were limited to clinical trials and which are anticipated to continue or added in clinical practice.

### 2.3 Semistructured interviews

Semistructured interviews were conducted from March 1 2024, to August 31, 2024 to validate the compiled list of care activities identified from SMPCs and trial protocols. Furthermore, these interviews aimed to gather insights into the prospective implementation of hemophilia GT in clinical practice. During these interviews, participants were first invited to evaluate the preliminary process map and determine whether the depicted care activities aligned with their experiences in clinical trials. Next, they were prompted to identify the activities that differ between GT for hemophilia A and B. Lastly, participants were asked to reflect on whether resources identified for hemophilia A and B in a clinical trials setting would change when GTs transition to clinical practice setting. If any changes were anticipated by the experts, they were requested to specify these discrepancies. The likelihood and validity of these changes were confirmed via triangulation, until data saturation was reached [28,29].

Eligible participants were healthcare professionals involved in GT delivery in clinical trials, including hemophilia doctors, hemophilia nursing professionals/practitioners, hospital/research pharmacists, and environmental safety officers. Participants were included from 4 hemophilia treatment centers (HTCs) in the Netherlands, namely: Erasmus Medical Center, Amsterdam University Medical Center, University Medical Center Utrecht, and University Medical Center Groningen. Purposive sampling was used to select participants. Interviews were conducted in person or online using a predefined topic guide. The interviews lasted ∼60 minutes, were audio-recorded, transcribed, and analyzed.

### 2.4 Resource identification and cost allocation

Cost allocation involved identifying resources for each activity and matching them with available cost data [30]. Each activity on the process map was assessed to identify the resources needed to carry out the activity. These resources were split up in 3 categories: personnel, materials, or infrastructure. For most activities, lump-sum cost data were available. For example, a lump-sum cost was available for consultations with medical specialists, which was assumed to cover all associated resources, including personnel, facilities, and office equipment [31].

Main sources for cost data were the reference prices from the Dutch Costing Manual and tariffs from the Dutch Healthcare Authority [31,32]. For activities related to environmental safety, cost data were available from local guideline [33]. For consumables, unit costs were obtained from relevant literature complemented with market prices [34,35]. For drugs, list prices from the Dutch National Health Institute were used [36]. Literature was searched in case cost data were unavailable from the previously mentioned sources. If no identical resource was found, assumptions were made based on similar resources and validated with experts (healthcare professionals with knowledge in costs, excluding those directly interviewed). Activity-based costing was used as a last measure when data were unavailable from all potential sources [30].

Identified costs were converted to Dutch 2024-unit costs in Euros (€) using healthcare-specific inflation rates provided by the Dutch Healthcare Authority [32]. Costs in other currencies than Euros were converted using Purchasing Power Parities data from the Organization for Economic Co-operation and Development[37].

### 2.5 Cost analyses

Costs calculated in this study were presented by treatment phase and treatment scenario, with separate cost estimations for hemophilia A and hemophilia B. Costs by phase included a one-time site preparation cost per hospital, as well as individual costs for each phase, including the second-year follow-up. Costs by treatment scenario represented the total cost covering all treatment phases. For individuals deemed noneligible for GT, only the direct costs associated with the screening phase were considered. For eligible individuals, direct costs related to screening, GT administration, and subsequent follow-up were included, along with indirect costs associated with pretreatment preparation. For the subset of eligible individuals who developed liver function abnormalities, additional management costs were included, covering corticosteroid treatment as well as the associated expenses for additional medications and monitoring required to address corticosteroid-related side effects [38,39]. We assumed that corticosteroid treatment to manage liver function abnormalities lasted for 242.9 days for hemophilia A and 79.8 days for hemophilia B [38,40,41]. For all types of costs, the mean values were calculated according to the treatment pathway or care processes that interviewees considered to be the most common.

Furthermore, sensitivity analyses were performed in which minimum and maximum costs were defined for each activity. Sensitivity analyses were conducted to assess how variations in resource use and cost inputs impact the total cost calculation. Sources of cost variation differed based on the care activity and data availability, including factors such as time variations and differences in the number of consultations. In the absence of data, a ± 20% range was applied to introduce cost variations in line with the Dutch health economic guideline [31]. All calculations were performed using Microsoft Excel (Microsoft).

### 2.6 Ethical approval

All participants were informed about the study’s purpose and methods and provided consent for the use of their interview data. The medical ethics review committee of Erasmus Medical Center assessed whether this research is subject to the Medical Research Involving Human Subjects Act and determined that it is not (medical ethics review committee number: MEC-2023-0799).

## 3 Results

### 3.1 Health care resources

Fifteen interviews with healthcare professionals were conducted (4 doctors, 4 nursing professionals, 4 pharmacists, and 3 environmental safety officers). Through these interviews we identified 5 phases in delivering hemophilia GTs in a clinical practice: site preparation, screening, pretreatment preparation, administration day, and follow-up (Figure). Experts expected that resources used were similar for both hemophilia A and hemophilia B. The primary distinction lies in the frequency and activities during the follow-up phase. For example, intensive follow-up, specifically of the liver function test, is required weekly for 26 weeks in hemophilia A, compared with weekly for 12 weeks in hemophilia B. A detailed overview of all identified resources and costs per phase are included in Supplementary Table 1 (hemophilia A) and Supplementary Table 2 (hemophilia B).

**Figure:**
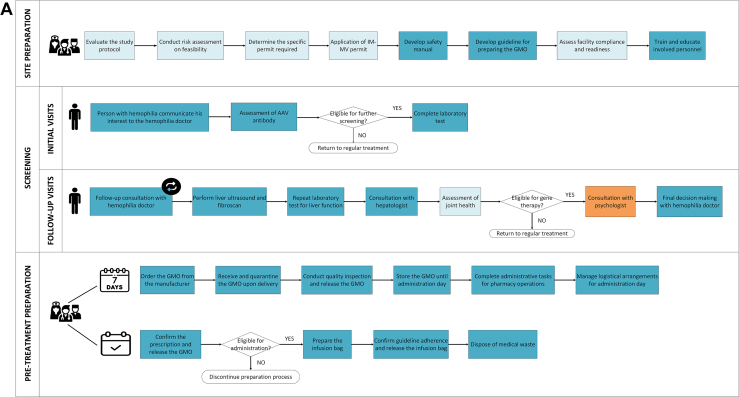

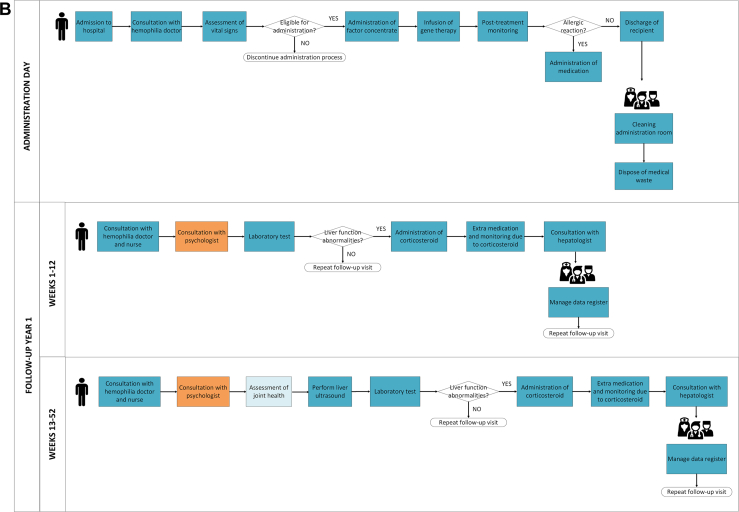

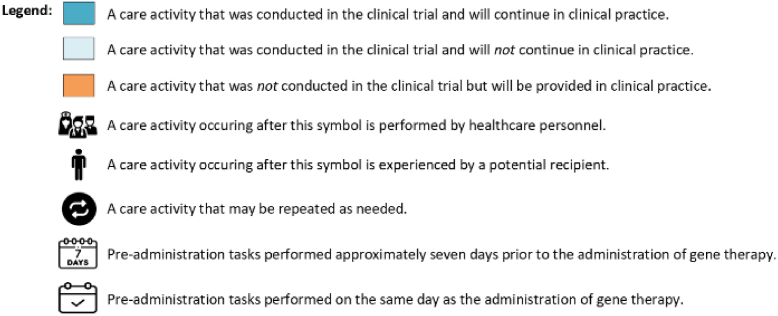
Process map illustrating the care delivery of gene therapy for hemophilia A and B. AAV, adeno-associated virus; GMO, genetically modified organisms; IM-MV, Environment-Medical Veterinary Research.

The site preparation phase ensures that sites are fully equipped for GT delivery. A key activity in clinical trials was obtaining the Environment-Medical Veterinary Research permit, managed by environmental safety officers. Required resources in clinical trials included labor from environmental safety officers, pharmacists, pharmacy technicians, doctors, and nurses. While these resources will still be needed in clinical practice, their quantities will vary due to a reduction in the time and effort required from involved personnel. For example, the workload for environmental safety officers and pharmacists is expected to decline, as preparatory tasks such as permit applications are presumed completed during the clinical trial phase.

The screening phase determines whether individuals with hemophilia interested in GT meet the eligibility criteria. This phase involves a multidisciplinary team including a hemophilia doctor, nurse, hepatologist, psychologist, and physical therapist (Figure). In clinical practice, this phase is expected to begin with AAV antibody testing, which constitutes the initial exclusion point. For hemophilia A, a positive result leads to exclusion, whereas a negative result allows progression to further laboratory assessments. For hemophilia B, although a positive result is not an exclusion criterion, testing is still performed to establish a baseline measurement. The second exclusion point is reached following the completion of all laboratory and liver function assessments, when eligibility is determined based on the overall clinical findings. Required resources are expected to include multiple outpatient visits with the multidisciplinary team, laboratory tests, and liver function assessments. Resource use in this phase is expected to differ slightly between clinical trials and clinical practice. Based on interviews, joint assessments by a physiotherapist—conducted in clinical trials to meet specific endpoints—are not expected to be routinely performed in clinical practice. However, such assessments may still be warranted on a case-by-case basis, particularly for patients with preexisting hemophilic arthropathy. Conversely, psychological consultations, which were not part of the clinical trials, are expected to be included in clinical practice.

The pretreatment preparation phase consists of 2 stages, both designed to ensure the necessary resources are in place for the day of administration. The first stage will take place approximately 7 days before GT administration and includes acquiring the GT product. The second stage will involve preparing the infusion bag containing the GT for administration on the designated day. The expected resources for this phase are similar between clinical trials and clinical practice, including personnel, hospital facilities, and materials required for infusion preparation.

On the day of administration, GT recipients are admitted to the hospital for infusion. The key activities during this phase will include the infusion process and postinfusion monitoring for potential allergic reactions. In clinical trials, some HTCs performed infusions in the nursing ward of the clinical department, while others used dedicated spaces for short inpatient stays. In clinical practice, each HTC will need to determine the appropriate space for administering GT infusion. The resources required are expected to align with those used in clinical trials, including inpatient facilities, physician and nursing care, materials for intravenous administration, and medications to manage allergic reactions.

The follow-up phase focuses on evaluating the effectiveness of hemophilia GT by assessing factor levels, frequency of bleeding episodes, and potential side effects, including liver function abnormalities. Key activities during this phase will include regular consultations and laboratory tests, with hospital visit frequency varying by hemophilia type and over time. In clinical trials, the follow-up period lasted from 5 to 15 years depending on the vector and regulatory jurisdiction. Based on interviews, follow-up in clinical practice requires to last at least 15 years or possibly a lifetime in clinical practice. Each visit will require resources for doctor and nursing consultations, laboratory tests, and data collection. In case liver abnormalities arise, additional resources are needed such as corticosteroid treatment, medications and monitoring for its side effects, and hepatologist consultations. As in the screening phase, psychological consultations will be incorporated into clinical practice, while joint health assessments will be excluded. Joint assessment is not required in the context of GT treatment. However, hemophilia doctors indicated that such assessments will be conducted when GT recipients continue to experience joint problems resulting from previous bleeding episodes, as these preexisting conditions are not expected to resolve following GT.

### 3.2 Costs

#### 3.2.1 Site preparation costs

In the interviews we identified preparatory activities that will be needed in real-world clinical practice. These include developing safety manuals, updating genetically modified organism preparation guidelines, and training personnel. Our analysis estimated this cost at €10,409 (€5305-€12,889) (Table 1). There is no difference in site preparation costs between hemophilia A and B. The largest cost driver is personnel training, estimated at €6656 (64%). Of this amount, 88% is allocated for training pharmacy technicians, conducted by hospital pharmacists, while the remaining 12% is designated for training nurses, conducted by hemophilia doctors. Based on interviews, training pharmacy technicians requires 24 hours of combined time for both the trainer and trainee, and nurses require 2 hours. The second-largest cost is labor costs for environmental safety officers, estimated at €3500 (34%). The remaining 2% accounts for hospital pharmacists’ labor costs related to updating genetically modified organism preparation guidelines to align with clinical practice.

**Table 1:** Estimated mean cost per individual by treatment scenario in real-world clinical practice.

Scenario	Hemophilia A	Hemophilia B
Noneligible individual after completing screening process	€2093 (€1723-€5152)	€2098 (€1736-€5111)
Eligible individual who remained free of liver function abnormalities during follow-up	€28,696 (€20,873-€48,973)	€20,511 (€18,175-€36,310)
Eligible individual who developed liver function abnormalities during follow-up	€29,460 (€21,485-€49,890)	€20,807 (€18,412-€36,665)

A comparison of site preparation costs between clinical practice and clinical trial is presented in Supplementary Table 1 for hemophilia A and Supplementary Table 2 for hemophilia B. Although site preparation costs for clinical trials are presented as supplementary, interview findings suggested that the actual resource requirements for delivering hemophilia GT in clinical trials are significantly greater, leading to higher overall costs. For instance, clinical trials incurred additional expenses not captured in our analysis, including ethical approval by institutional review boards, study start-up fees, trial management, support staff (eg, sample processing and shipment to central laboratory), document filing, and updates to electronic case report forms.

#### 3.2.2 Delivery costs

##### 3.2.2.1 Noneligible individual after completing screening process

Individuals deemed noneligible following AAV antibody testing (Figure) incur a one-time cost of €151, which includes a doctor consultation and the AAV antibody test. This cost is identical for both hemophilia A and B. For individuals who complete the full screening process but are subsequently deemed ineligible, the incurred screening costs are equivalent to those of eligible individuals. The total screening cost is estimated at €2093 (€1723-€5152) for hemophilia A and €2098 (€1736-€5111) for hemophilia B (Table 1).

##### 3.2.2.2 Eligible individual who remained free of liver function abnormalities during follow-up

The estimated total cost for an eligible individual who remains free of liver function abnormalities during the follow-up period is €28,696 (€20,873-€48,973) for hemophilia A and €20,511 (€18,175-€36,310) for hemophilia B (Table 1). These totals include costs related to screening, pretreatment preparation, administration, and the first-year follow-up. In both hemophilia A and hemophilia B, the first-year follow-up cost is the primary cost driver. For hemophilia A, costs are distributed as follows: 7% for screening (€2093), 9% for pretreatment preparation (€2602), 6% for administration (€1754), and 78% for first-year follow-up (€22,247). For hemophilia B, the distribution is 10% for screening (€2098), 13% for pretreatment preparation (€2602), 8% for administration (€1754), and 69% for the first-year follow-up (€14,058) (Table 2).

**Table 2:** Estimated mean cost per individual by phase in real-world clinical practice.

Phase	Hemophilia A	Hemophilia B
Site preparation^a^	€10,409 (€5305-€12,889)	€10,409 (€5305-€12,889)
Screening	€2093 (€1723-€5152)	€2098 (€1736-€5111)
Pretreatment preparation	€2602 (€1576-€4637)	€2602 (€1576-€4637)
Administration day	€1754 (€1550-€3882)	€1754 (€1550-€3882)
Follow-up^b^		
Year 1	€22,247 (€16,025-€35,302)	€14,058 (€13,313-€22,680)
Year 2	€2633 (€1447-€2863)	€1694 (€977-€2567)

a The costs of site preparation were a one-time charge per hospital.

b Follow-up costs did not include expenses associated with liver function abnormalities.

The most significant cost difference between hemophilia A and B occurs during the follow-up phase, as individuals with hemophilia A will require more frequent hospital visits due to occurrences of liver function abnormalities. Individuals with hemophilia A are expected to attend weekly hospital visits during the first 6 months, followed by biweekly visits for the remainder of the year. In contrast, individuals with hemophilia B are expected to have weekly visits for the first 12 weeks, followed by monthly visits thereafter. This difference in visit schedules results in a €8189 (€2698-€12,663) higher follow-up cost for hemophilia A compared with hemophilia B.

The second-year follow-up cost in clinical practice was also estimated. For hemophilia A, the estimated cost is €2633 (€1447-€2863) per individual, based on 4 hospital visits per year. In clinical trials, visit frequency for hemophilia A decreased to 2 visits per year from the third year onward, which corresponds to an annual cost of approximately €1521 if adopted in clinical practice (Supplementary Table 1). For hemophilia B, the second-year follow-up cost is estimated at €1694 (€977-€2567) per individual, based on 2 visits per year. Following clinical trial experience, from the third year onward, follow-up is expected to decline to one annual visit for hemophilia B, with an associated cost of ∼€1051 per individual per year (Supplementary Table 2). However, interview findings suggest that visit frequency may vary depending on the duration of FVIII or FIX expression in each individual.

##### 3.2.2.3 Eligible individual who developed liver function abnormalities during follow-up

Estimates were also made for individuals who were eligible, received treatment but developed liver function abnormalities during follow-up. For hemophilia A, the additional cost of managing liver function abnormalities is estimated at €764 (€612-€917), comprising corticosteroid treatment, medications and monitoring for its side effects, and hepatologist consultations (Supplementary Tables 1 and 2). This increases the total estimated cost to €29,460 (€21,485-€49,890). For hemophilia B, the cost for managing liver function abnormalities is estimated at €296 (€237-€355), resulting in a total cost of €20,807 (€18,412-€36,665) for affected individuals. The difference in management costs between hemophilia A and B is attributable to the longer duration of treatment for liver function abnormalities in hemophilia A (242.9 days) compared with hemophilia B (79.8 days).

## 4 Discussion

This microcosting study aimed to estimate the health care resources and costs of delivering GT for hemophilia A and B in clinical practice. We found that the delivery of GT for hemophilia requires substantial resources, primarily concentrated within the first year of treatment. These include increased personnel, more frequent hospital visits, laboratory tests, liver function assessments, medications, medical consumables, and hospital facilities and equipment. Resources for GT delivery are identified across 5 phases: site preparation, screening, pretreatment preparation, administration day, and follow-up, and visualized in a process map.

Implementing hemophilia GT in clinical practice involves a one-time site preparation cost per hospital, estimated at €10,409 (€5305-€12,889). The cost incurred for individuals excluded after initial screening is €151 per individual, consistent across both hemophilia A and B. For those advancing to complete screening but subsequently deemed noneligible, the estimated individual costs are €2093 (€1723-€5152) for hemophilia A and €2098 (€1736-€5111) for hemophilia B. The estimated total cost in clinical practice for an eligible individual without liver function abnormalities, including screening, pretreatment preparation, administration, and first-year follow-up, is €28,696 (€20,873-€48,973) for hemophilia A and €20,511 (€18,175-€36,310) for hemophilia B. In cases where GT recipients develop liver function abnormalities during the first-year follow-up, the estimated total cost increases to €29,460 (€21,485-€49,890) for hemophilia A and €20,807 (€18,412-€36,665) for hemophilia B. The estimated individual costs for second-year follow-up are €2633 (€1447-€2863) for hemophilia A and €1694 (€977-€2567) for hemophilia B.

Individuals with hemophilia are known to be well-trained to administer their standard treatment (factor or nonfactor therapies) at home [4,42]. They may visit the hospital only once or twice a year for check-ups or in case of severe bleeding episodes, which require minimal in-hospital care. In contrast, our findings show that delivering GTs for hemophilia initially demands significantly more hospital-based care, particularly on the day of administration and during follow-up visits. This raises important questions about the long-term impact of implementing GTs and how the introduction of additional GT products, both for hemophilia and other conditions, may affect hospital care structures in terms of organization. As our understanding of GT delivery advances and more individuals are treated, resource demands may decrease over time due to increased process efficiency and learnings. However, whether this reduction will match the resource requirements of current prophylactic treatments remains to be seen.

Guidance documents for implementing GT for hemophilia have been published for several countries, including Italy, the United Kingdom, the United States, and the Nordic region [[10], [11], [12], [13]]. These documents outline necessary resources, such as specialized infrastructure, a multidisciplinary care team, and laboratory tests. However, they do not quantify these resources and their associated costs. For example, Astermark et al. [18] detail procedures for the pre-, peri-infusion, and postinfusion stages in the Nordic context but do not specify the frequency of resource use. This study addresses this gap by specifically quantifying the frequency of resource use per unit and estimating the associated costs.

The resource demands for implementing hemophilia GT may vary depending on the circumstances of the setting in which this therapy is delivered. In this study, we focused on centers with prior experience in GT during clinical trials and that are expected to provide both infusion and follow-up in clinical practice. Consequently, many of the necessary resources may already be available at these centers, reducing the need to establish processes from the outset. By contrast, centers without prior experience may need to organize all care activities de novo and therefore require additional resources. Furthermore, centers may function solely as infusion centers, follow-up centers, or both, which implies that resource requirements must be tailored to their specific context. Such tailored calculations can be performed using the comprehensive resource catalog provided in this study (Supplementary Tables 1–4), which enables centers to identify the required resources, align them with local cost units, and estimate the costs of GT implementation in their own setting.

The information on resource use is useful not only for guiding GT implementation but also for economic evaluations and reimbursement discussions. A few (early) economic evaluations have assessed the cost-effectiveness of GT compared with standard care [7,[16], [17], [18], [19], [20]]. However, none of these accounted for all the resources required for GT delivery, as outlined in this study. For example, in their early economic evaluation of GT for hemophilia A, ten Ham et al. [7] included only healthcare costs related to medications, bleed-related and nonbleed-related events, surgery, and adverse events. This approach likely underestimates the total costs of GT delivery by excluding essential components of the care process, including screening, administration, and follow-up. Only Bolous et al. [17] incorporated most of identified resources into their cost-effectiveness analysis, although with varying frequencies. Notably, pretreatment preparation costs such as labor costs for hospital pharmacists and nurses were not included. Nevertheless, Bolous et al. [17] demonstrated that GT for hemophilia B remained cost-effective when delivery costs were considered. The impact of delivery costs on the overall cost-effectiveness of GT for hemophilia A requires further investigation but may be more dependent upon the price of the GT itself.

Our cost estimations for GT delivery in clinical practice included the cost of psychological consultations [17]. Hemophilia specialists noted that in the trials GT recipients did not exhibit signs of psychological issues that would warrant such consultations. However, there is ongoing debate whether psychological consultations should be offered to potential GT recipients [[43], [44], [45], [46], [47]]. Given that GT is a permanent and transformative treatment, it may affect the recipient's sense of identity [47]. Furthermore, recognition of the potential risks, such as treatment failure and long-term complications, highlights the need to consider the associated psychological impacts in clinical practice. As a result, this study acknowledges the importance of psychosocial support and includes psychologist consultations as a new resource in clinical practice, leading to slightly higher estimated costs. While this approach may slightly overestimate costs in clinical practice, incorporating psychological consultations into the GT care pathway ensures both accessibility and potential reimbursement. When involving psychologists in clinical practice, it is important to consider that they may not be specifically trained to support GT recipients and may therefore require additional training. The scope of such training should be determined collaboratively by the relevant healthcare professionals, after which the associated additional costs can be assessed.

The health care resources and estimated costs identified in this study provide a starting point for stakeholders to initiate reimbursement discussions. Given that hemophilia care in the Netherlands has traditionally been predominantly home-based, the increased reliance on hospital services necessitated by GTs may require a revision of existing budgeting and payment structures. Therefore, in addition to the cost of the GT products, the costs associated with delivering the therapy itself must be considered. Although the delivery costs are significantly lower than the GT product costs, the estimated range of €20,511 (hemophilia B) to €28,696 (hemophilia A) remains substantial and may place additional financial strain on already limited hospital and healthcare budgets.

Discussions regarding reimbursement should also address variations in care consumption and costs between hemophilia A and B, as well as across potential categories of GT recipients. This study highlights that noneligible individuals also incurred costs, suggesting the need to specifically account for this group in reimbursement agreements. Furthermore, individuals who develop liver function abnormalities during follow-up, which are more common in hemophilia A [38,39], tend to incur higher costs than those without. Therefore, this group should also be considered in related agreements. Notably, liver function abnormalities in this study were defined as elevations in alanine aminotransferase levels observed during the 2-year follow-up period. Such abnormalities may not only reflect transient immune-mediated responses but could also signal delayed hepatologic complications. These potential liver-related complications were not incorporated into the present analysis but warrant consideration in future evaluations.

Despite our efforts, this study has several limitations. First, this study did not have access to individual-level resource utilization data from clinical practice, as hemophilia GT is not yet implemented in routine care in the Netherlands. As a result, estimates of mean costs were based on assumed standard care pathways rather than on empirically derived averages from actual data. However, these assumptions were validated through expert interviews to ensure they reasonably reflect real-world clinical practice. Second, this study did not account for whether specific laboratory assessments would be conducted in a central laboratory. In clinical trials, some tests were performed in a central laboratory. Interview findings suggest that in clinical practice only the AAV test will be conducted centrally as other laboratory assessments will likely be carried out at the hospital level. Consequently, costs associated with central AAV testing such as sample shipment, handling, and coordination were not included in the analysis. Third, the current analysis was limited to the interval from site preparation through 2 years of follow-up and focused primarily on nonpharmaceutical costs related to GT delivery. Similar to patients receiving prophylaxis, GT recipients may still require supplementary factor concentrate in cases of trauma or medical procedures, which could influence long-term healthcare costs. Therefore, the costs estimated in this study should be supplemented with pharmaceutical-related costs to enable a comprehensive assessment of long-term costs and ultimately the long-term cost-effectiveness of GT. Last, our findings should not be assumed to be fully representative of other types of GTs for hemophilia or beyond. The care activity data for this study was derived from the BMN-270, AMT-060, and AMT-061 trials. Therefore, the identified costs in this study are specific to the resources used in these trials and the corresponding AAV-based GTs. Certain elements may be transferable to other GTs or indications, yet thorough assessment is needed. When (hemophilia) GTs differ significantly from those examined in this study [48,49], future research could adopt the same methodology used here to estimate resources and costs for these new GTs and settings.

Regardless of the limitations, the comprehensive list of health care resources identified in this study is considered one of its key strengths. The information on the resources required to deliver hemophilia GTs can serve as a reference for other (inter)national hospitals. While this study utilized Dutch unit costs, the resource utilization data presented can be adapted by applying country-specific costs to estimate the total cost associated with delivering hemophilia GTs in other countries or settings.

### 4.1 Future directions

This study provides insights on 3 levels. First, it highlights the shift in hemophilia care associated with GT delivery from outpatient to inpatient care, driven by the increased demand for hospital-based services during the screening, administration, and follow-up phases. As more GT products enter the market for hemophilia, it becomes essential to evaluate not only their short-term and long-term clinical outcomes but also their broader implications, including required changes in care delivery and health system organization both now and in the future. Second, the identified resources can serve as inputs for future cost-effectiveness analyses. We encourage future research to include, in addition to GT price itself, all identified resources in their analyses and to assess their influence on the overall cost-effectiveness outcomes. Future cost-effectiveness analyses should also consider costs associated with clinical outcomes, such as post-GT bleeding in patients whose factor levels remain within the mild-to-moderate hemophilia range or potential resumption of prophylaxis due to loss of efficacy. These aspects were not included in the present analysis but may influence overall economic outcomes. Last, our study provides cost insights to inform reimbursement discussions on care delivery of valoctocogene roxaparvovec and etranacogene dezaparvovec. These discussions may consider variations in care consumption among potential GT recipients and the potential range of associated costs. As only a limited number of individuals have received hemophilia GT outside clinical trials, real-world data on health care resource use remain scarce. Therefore, it is recommended to systematically document real-world resource utilization and to reassess costs accordingly after implementation of GT for hemophilia in clinical practice.
