# Supplementary material for: Health care resources and costs associated with delivering gene therapy for hemophilia in clinical practice
Source: Res Pract Thromb Haemost. 2025 Dec 2;10(1):103275. doi: 10.1016/j.rpth.2025.103275 (PMC12800355; doi:10.1016/j.rpth.2025.103275)
Supplement: Supplementary Tables 1–4 [file mmc1.docx]

**Electronic Supplementary Materials**

**Health care resources and costs associated with delivering gene therapy for hemophilia in clinical practice**

**Diaz M. Prameyllawati^1,2^, Caroline M.A. Mussert^3^, Martijn A.H. Oude Voshaar^1^, Hester F. Lingsma^1^, Marjon H. Cnossen^3^, Michiel Coppens^4,5^, Karina Meijer^6^, Paul R. van der Valk^7^, Frank W.G. Leebeek^8^, Renske M.T. ten Ham^2^**

^1^Department of Public Health, Erasmus MC, University Medical Center Rotterdam, Rotterdam, the Netherlands

^2^Department of Epidemiology and Health Economics, Julius Center for Health Sciences and Primary Care, University Medical Center Utrecht, Utrecht, the Netherlands

^3^Department of Pediatric Hematology and Oncology, Erasmus MC Sophia Children’s Hospital, University Medical Center Rotterdam, Rotterdam, the Netherlands

^4^Department of Vascular Medicine, Amsterdam University Medical Center, University of Amsterdam, Amsterdam, the Netherlands

^5^Amsterdam Cardiovascular Sciences, Pulmonary Hypertension & Critical Care, Amsterdam, the Netherlands

^6^Department of Hematology, University Medical Center Groningen, University of Groningen, Groningen, the Netherlands

^7^Center for Benign Hematology, Thrombosis and Hemostasis, Van Creveldkliniek, University Medical Center Utrecht, Utrecht University, Utrecht, the Netherlands

^8^Department of Hematology, Erasmus MC, University Medical Center Rotterdam, Rotterdam, the Netherlands

**Corresponding Author:**

Diaz M. Prameyllawati, MSc

d.prameyllawati@erasmusmc.nl

Department of Public Health

Erasmus MC, University Medical Center Rotterdam

Rotterdam, The Netherlands

**Table 1** Breakdown of resources used and associated costs for delivering gene therapy for hemophilia A

| **Care activity (hospital perspective)** | **Frequency per unit** | **Clinical trial** | **Clinical practice** | **Source** |
| --- | --- | --- | --- | --- |
| **Site preparation** |  | **€ 22,562** | **€ 10,409** |  |
| Labor cost of ESO |  | € 15,400 | € 3,500 |  |
| Determine the specific permit required* | One-time charge per center | € 3,500 |  | (1) |
| Application of IM-MV permit* | One-time charge per center | € 4,200 |  | (1) |
| Maintenance of IM-MV module throughout the trial period* | One-time charge per center | € 4,200 |  | (1) |
| Develop safety manual | One-time charge per center | € 3,500 | € 3,500 | (1) |
| Labor cost of hospital pharmacist |  | € 506 | € 253 |  |
| Evaluate study protocol* | One-time charge per center | € 253 |  | ABC for 2 hours |
| Develop guideline for preparing the GMO | One-time charge per center | € 253 | € 253 | ABC for 2 hours |
| Train and educate involved personnel |  | € 6,656 | € 6,656 |  |
| Handling and preparation of the GMO  (1 hospital pharmacist and 2 pharmacy technicians) | One-time charge per center | € 5,853 | € 5,853 | ABC for 24 hours |
| Knowledge about GT and infusion process  (2 hemophilia doctors, 1 nurse specialist, 1 nurse consultant) | One-time charge per center | € 803 | € 803 | ABC for 2 hours |
| **Screening** |  | **€ 1,999** | **€ 2,093** |  |
| Consultation with healthcare professionals |  | € 975 | € 1,069 |  |
| Consultation with hemophilia doctor | Three consultations per individual | € 411 | € 411 | (2) |
| Consultation with nurse specialist | Three consultations per individual | € 411 | € 411 | (2) |
| Consultation with hepatologist | One consultation for every 8 out of 10 individuals | € 110 | € 110 | (2) |
| Consultation with psychologist** | One consultation per individual |  | € 138 | (2) |
| Consultation with physical therapist (assessment of joint health)* | One consultation per individual | € 44 |  | (2) |
| Complete lab test |  | € 755 | € 755 |  |
| AAV5 diagnostic test | One test per individual | € 14 | € 14 | (3) |
| Blood count: Hb, HCT,  thrombocyte, leucocyte, differential | One test per individual | € 9 | € 9 | (4) |
| Hemostasis: INR, PT, APTT,  fibrinogen, d-dimer | One test per individual | € 32 | € 32 | (4) |
| Factor VIII activity level (one-stage assay) | One test per individual | € 16 | € 16 | (4) |
| Factor VIII activity level (chromogenic assay) | One test per individual | € 58 | € 58 | (4) |
| Factor VIII antigen | One test per individual | € 51 | € 51 | (4) |
| Factor VIII inhibitor | One test per individual | € 9 | € 9 | Assumed to have an equal cost that of factor IX inhibitor |
| Liver function: AST, ALT, LDH, ALP,  GGT, total bilirubin, albumin | Two tests per individual | € 29 | € 29 | (2, 4) |
| Renal function: creatinine, urea | One test per individual | € 4 | € 4 | (2, 4) |
| Virology: hepatitis serology,  HBsAg, HBV DNA, anti-HCV,  HCV RNA, HIV serology | One test per individual | € 490 | € 490 | (4) |
| Tumor marker: AFP, PIVKA-II | One test per individual | € 36 | € 36 | (4) |
| Inflammatory marker: CRP, ESR | One test per individual | € 7 | € 7 | (4) |
| Liver function assessment |  | € 269 | € 269 |  |
| Liver ultrasound | One assessment per individual | € 135 | € 135 | (2) |
| FibroScan | One assessment per individual | € 135 | € 135 | (2) |
| **Pre-treatment preparation** |  | **€ 2,602** | **€ 2,602** |  |
| Labor cost of hospital pharmacist |  | € 295 | € 295 |  |
| Order process of the GMO | One occurrence per individual | € 42 | € 42 | ABC for 0.3 hour |
| Receive and quarantine the GMO upon delivery | One occurrence per individual | € 63 | € 63 | ABC for 0.5 hour |
| Conduct quality inspection and release the GMO | One occurrence per individual | € 63 | € 63 | ABC for 0.5 hour |
| Complete administrative tasks for pharmacy operations | One occurrence per individual | € 127 | € 127 | ABC for 1 hour |
| Labor cost of nurse specialist |  | € 1,808 | € 1,808 |  |
| Manage logistical arrangements for administration day | One occurrence per individual | € 1,808 | € 1,808 | ABC for 26 hours |
| Preparation of gene therapy infusion |  | € 498 | € 498 |  |
| *Labor cost of hospital pharmacist* |  | € 75 | € 75 |  |
| Confirm the prescription and release the GMO | One occurrence per individual | € 25 | € 25 | ABC for 0.25 hour |
| Confirm guideline adherence and release the infusion bag | One occurrence per individual | € 50 | € 50 | ABC for 0.5 hour |
| *Labor cost of pharmacy technician* |  | € 185 | € 185 |  |
| Prepare the cleanroom facility | One occurrence per individual | € 46 | € 46 | ABC for 0.5 hour |
| Actual preparation work by two pharmacy technicians | One occurrence per individual | € 92 | € 92 | ABC for 1 hour |
| Clean the cleanroom facility after preparation | One occurrence per individual | € 46 | € 46 | ABC for 0.5 hour |
| *Materials used for preparation* | One set of material per individual | € 133 | € 133 | (5, 6) |
| *Storage and cleanroom facilities for GMO, and GMO waste management* | One occurrence per individual | € 106 | € 106 | Overhead costs (calculated as 27% of the total preparation cost) |
| **Administration day** |  | **€ 1,754** | **€ 1,754** |  |
| Admission to inpatient facility including nursing care, room cleaning and medical waste disposal | One occurrence per individual | € 449 | € 449 | (2) |
| Consultation with hemophilia doctor | One consultation per individual | € 137 | € 137 | (2) |
| Infusion of gene therapy |  | € 345 | € 345 |  |
| Intravenous administration | One occurrence per individual | € 195 | € 195 | (2) |
| Presence of hemophilia doctor during the infusion | One occurrence per individual | € 150 | € 150 | ABC for 1 hour |
| Labor cost for post-treatment follow-up  (1 hemophilia doctor and 1 nurse specialist) | One occurrence per individual | € 820 | € 820 | ABC for 4 hours |
| Drug costs |  | € 3 | € 3 |  |
| Administration of medication for allergic reaction (adrenaline) | One administration for every 1 out of 10 individuals | € 3 | € 3 | (7) |
| **Follow-up Year 1**^†^ |  | **€ 22,824** | **€ 23,011** |  |
| **Year 1 (Weeks 1-26)** |  | **€ 15,153** | **€ 15,247** |  |
| Consultation with hemophilia doctor | 26 consultations per individual | € 3,559 | € 3,559 | (2) |
| Consultation with nurse specialist | 26 consultations per individual | € 3,559 | € 3,559 | (2) |
| Consultation with psychologist** | One consultation per individual |  | € 138 | (2) |
| Consultation with physical therapist (assessment of joint health)* | One consultation per individual | € 44 |  | (2) |
| Lab test   - Blood count: Hb, HCT, thrombocyte, leucocyte, differential - Hemostasis: INR, PT, APTT, fibrinogen, d-dimer - Factor VIII: one-stage and chromogenic factor activity level, antigen, inhibitor - Liver function: AST, ALT, LDH, ALP, GGT, total bilirubin, albumin | 26 tests per individual | € 4,938 | € 4,938 | (2, 4, 8) |
| Administration of medication for liver function abnormalities^††^   - Corticosteroid: prednisolone 60mg - Extra medication: calcium carbonate with vitamin D, omeprazole, cotrimoxazole - Extra follow-up: glucose, hypertension | 183-day course of corticosteroid treatments per individual | € 369 | € 369 | (4, 7) |
| Liver ultrasound | One assessment per individual | € 135 | € 135 | (2) |
| Consultation with hepatologist^††^ | One consultation per individual | € 137 | € 137 | (2) |
| Manage data register | 26 occurrences per individual | € 2,412 | € 2,412 | ABC for 2 hours per occurrence |
| **Year 1 (Weeks 27-52)** |  | **€ 7,671** | **€ 7,765** |  |
| Consultation with hemophilia doctor | 13 consultations per individual | € 1,779 | € 1,779 | (2) |
| Consultation with nurse specialist | 13 consultations per individual | € 1,779 | € 1,779 | (2) |
| Consultation with psychologist** | One consultation per individual |  | € 138 | (2) |
| Consultation with physical therapist (assessment of joint health)* | One consultation per individual | € 44 |  | (2) |
| Lab test   - Blood count: Hb, HCT, thrombocyte, leucocyte, differential - Hemostasis: INR, PT, APTT, fibrinogen, d-dimer - Factor VIII: one-stage and chromogenic factor activity level, antigen, inhibitor - Liver function: AST, ALT, LDH, ALP, GGT, total bilirubin, albumin | 13 tests per individual | € 2,469 | € 2,469 | (2, 4, 8) |
| Administration of medication for liver function abnormalities^††^   - Corticosteroid: prednisolone 60mg - Extra medication: calcium carbonate with vitamin D, omeprazole, cotrimoxazole - Extra follow-up: glucose, hypertension | 60-day course of corticosteroid treatments per individual | € 121 | € 121 | (4, 7) |
| Liver ultrasound | One assessment per individual | € 135 | € 135 | (2) |
| Consultation with hepatologist^††^ | One consultation per individual | € 137 | € 137 | (2) |
| Manage data register | 13 occurrences per individual | € 1,206 | € 1,206 | ABC for 2 hours per occurrence |
| **Year 2** |  | **€ 2,584** | **€ 2,633** |  |
| Consultation with hemophilia doctor | Four consultations per individual | € 548 | € 548 | (2) |
| Consultation with nurse specialist | Four consultations per individual | € 548 | € 548 | (2) |
| Consultation with psychologist** | One consultation per individual |  | € 138 | (2) |
| Consultation with physical therapist (assessment of joint health)* | Two consultations per individual | € 89 |  | (2) |
| Lab test   - Blood count: Hb, HCT, thrombocyte, leucocyte, differential - Hemostasis: INR, PT, APTT, fibrinogen, d-dimer - Factor VIII: one-stage and chromogenic factor activity level, antigen, inhibitor - Liver function: AST, ALT, LDH, ALP, GGT, total bilirubin, albumin | Four tests per individual | € 760 | € 760 | (2, 4, 8) |
| Administration of medication for liver function abnormalities   - Corticosteroid: prednisolone 60mg - Extra medication: calcium carbonate with vitamin D, omeprazole, cotrimoxazole - Extra follow-up: glucose, hypertension | Assumed liver function abnormalities resolved within the first year |  |  |  |
| Liver ultrasound | Two assessments per individual | € 269 | € 269 | (2) |
| Consultation with hepatologist | Assumed liver function abnormalities resolved within the first year |  |  |  |
| Manage data register | Four occurrences per individual | € 371 | € 371 | ABC for 2 hours per occurrence |

*A care activity that was conducted in the clinical trial and will *not* continue in real-world practice. **A care activity that was *not* conducted in the clinical trial but will be provided in real-world practice. ^†^The estimation of follow-up costs encompassed the expenses associated with managing liver function abnormalities. ^††^The costs for administering medication for liver function abnormalities and consultations with a hepatologist were €0 if the eligible individual remained free of liver function abnormalities during the follow-up period. *AAV5* indicates adeno-associated virus 5; *ABC,* activity-based costing; *AFP,* alpha-fetoprotein; *ALP,* alkaline phosphatase; *ALT,* alanine aminotransferase; *APTT,* activated partial thromboplastin time; *AST,* aspartate aminotransferase; *CRP,* C-reactive protein; *DNA,* deoxyribonucleic acid; *ESO,* environmental safety officer; *ESR,* erythrocyte sedimentation rate; *GGT*, gamma-glutamyl transferase; *GMO,* genetically modified organisms; *Hb,* hemoglobin; *HBsAg,* hepatitis B surface antigen; *HBV*, hepatitis B virus; *HCT,* hematocrit; *HCV*, hepatitis C virus; *HIV*, human immunodeficiency virus, *IM-MV,* Environment-Medical Veterinary Research; *INR,* international normalized ratio; *LDH,* lactate dehydrogenase; *NZA,* Nederlandse Zorgautoriteit; *PIVKA-II,* protein induced by vitamin K absence-II, *PT*, prothrombin time; *RNA,* ribonucleic acid; *ZIN,* Zorginstituut Nederland.

**Table 2** Breakdown of resources used and associated costs for delivering gene therapy for hemophilia B

| **Care activity (hospital perspective)** | **Frequency per unit** | **Clinical trial** | **Clinical practice** | **Source** |
| --- | --- | --- | --- | --- |
| **Site preparation** |  | **€ 22,562** | **€ 10,409** |  |
| Labor cost of ESO |  | € 15,400 | € 3,500 |  |
| Determine the specific permit required* | One-time charge per center | € 3,500 |  | (1) |
| Application of IM-MV permit* | One-time charge per center | € 4,200 |  | (1) |
| Maintenance of IM-MV module throughout the trial period* | One-time charge per center | € 4,200 |  | (1) |
| Develop safety manual | One-time charge per center | € 3,500 | € 3,500 | (1) |
| Labor cost of hospital pharmacist |  | € 506 | € 253 |  |
| Evaluate study protocol* | One-time charge per center | € 253 |  | ABC for 2 hours |
| Develop guideline for preparing the GMO | One-time charge per center | € 253 | € 253 | ABC for 2 hours |
| Train and educate involved personnel |  | € 6,656 | € 6,656 |  |
| Handling and preparation of the GMO  (1 hospital pharmacist and 2 pharmacy technicians) | One-time charge per center | € 5,853 | € 5,853 | ABC for 24 hours |
| Knowledge about GT and infusion process  (2 hemophilia doctors, 1 nurse specialist, 1 nurse consultant) | One-time charge per center | € 803 | € 803 | ABC for 2 hours |
| **Screening** |  | **€ 2,004** | **€ 2,098** |  |
| Consultation with healthcare professionals |  | € 893 | € 987 |  |
| Consultation with hemophilia doctor | Three consultations per individual | € 411 | € 411 | (2) |
| Consultation with nurse specialist | Three consultations per individual | € 411 | € 411 | (2) |
| Consultation with hepatologist | One consultation for every 2 out of 10 individuals | € 27 | € 27 | (2) |
| Consultation with psychologist** | One consultation per individual |  | € 138 | (2) |
| Consultation with physical therapist (assessment of joint health)* | One consultation per individual | € 44 |  | (2) |
| Complete lab test |  | € 842 | € 842 |  |
| AAV5 diagnostic test | One test per individual | € 14 | € 14 | (3) |
| Blood count: Hb, HCT,  thrombocyte, leucocyte, differential | One test per individual | € 9 | € 9 | (4) |
| Hemostasis: INR, PT, APTT,  fibrinogen, d-dimer | One test per individual | € 32 | € 32 | (4) |
| Factor IX activity level (one-stage assay) | One test per individual | € 40 | € 40 | (4) |
| Factor IX activity level (chromogenic assay) | One test per individual | € 121 | € 121 | Assumed to have a cost three times that of factor IX activity level one-stage assay |
| Factor IX antigen | One test per individual | € 51 | € 51 | Assumed to have an equal cost that of factor VIII antigen |
| Factor IX inhibitor | One test per individual | € 9 | € 9 | (8) |
| Liver function: AST, ALT, LDH, ALP,  GGT, total bilirubin, albumin | Two tests per individual | € 29 | € 29 | (2, 4) |
| Renal function: creatinine, urea | One test per individual | € 4 | € 4 | (2, 4) |
| Virology: hepatitis serology,  HBsAg, HBV DNA, anti-HCV,  HCV RNA, HIV serology | One test per individual | € 490 | € 490 | (4) |
| Tumor marker: AFP, PIVKA-II | One test per individual | € 36 | € 36 | (4) |
| Inflammatory marker: CRP, ESR | One test per individual | € 7 | € 7 | (4) |
| Liver function assessment |  | € 269 | € 269 |  |
| Liver ultrasound | One assessment per individual | € 135 | € 135 | (2) |
| FibroScan | One assessment per individual | € 135 | € 135 | (2) |
| **Pre-treatment preparation** |  | **€ 2,602** | **€ 2,602** |  |
| Labor cost of hospital pharmacist |  | € 295 | € 295 |  |
| Order process of the GMO | One occurrence per individual | € 42 | € 42 | ABC for 0.3 hour |
| Receive and quarantine the GMO upon delivery | One occurrence per individual | € 63 | € 63 | ABC for 0.5 hour |
| Conduct quality inspection and release the GMO | One occurrence per individual | € 63 | € 63 | ABC for 0.5 hour |
| Complete administrative tasks for pharmacy operations | One occurrence per individual | € 127 | € 127 | ABC for 1 hour |
| Labor cost of nurse specialist |  | € 1,808 | € 1,808 |  |
| Manage logistical arrangements for administration day | One occurrence per individual | € 1,808 | € 1,808 | ABC for 26 hours |
| Preparation of gene therapy infusion |  | € 498 | € 498 |  |
| *Labor cost of hospital pharmacist* |  | € 75 | € 75 |  |
| Confirm the prescription and release the GMO | One occurrence per individual | € 25 | € 25 | ABC for 0.25 hour |
| Confirm guideline adherence and release the infusion bag | One occurrence per individual | € 50 | € 50 | ABC for 0.5 hour |
| *Labor cost of pharmacy technician* |  | € 185 | € 185 |  |
| Prepare the cleanroom facility | One occurrence per individual | € 46 | € 46 | ABC for 0.5 hour |
| Actual preparation work by two pharmacy technicians | One occurrence per individual | € 92 | € 92 | ABC for 1 hour |
| Clean the cleanroom facility after preparation | One occurrence per individual | € 46 | € 46 | ABC for 0.5 hour |
| *Materials used for preparation* | One set of material per individual | € 133 | € 133 | (5, 6) |
| *Storage and cleanroom facilities for GMO, and GMO waste management* | One occurrence per individual | € 106 | € 106 | Overhead costs (calculated as 27% of the total preparation cost) |
| **Administration day** |  | **€ 1,754** | **€ 1,754** |  |
| Admission to inpatient facility including nursing care, room cleaning and medical waste disposal | One occurrence per individual | € 449 | € 449 | (2) |
| Consultation with hemophilia doctor | One consultation per individual | € 137 | € 137 | (2) |
| Infusion of gene therapy |  | € 345 | € 345 |  |
| Intravenous administration | One occurrence per individual | € 195 | € 195 | (2) |
| Presence of hemophilia doctor during the infusion | One occurrence per individual | € 150 | € 150 | ABC for 1 hour |
| Labor cost for post-treatment follow-up  (1 hemophilia doctor and 1 nurse specialist) | One occurrence per individual | € 820 | € 820 | ABC for 4 hours |
| Drug costs |  | € 3 | € 3 |  |
| Administration of medication for allergic reaction (adrenaline) | One administration for every 1 out of 10 individuals | € 3 | € 3 | (7) |
| **Follow-up Year 1**^†^ |  | **€ 14,166** | **€ 14,353** |  |
| **Year 1 (Weeks 1-12)** |  | **€ 8,017** | **€ 8,155** |  |
| Consultation with hemophilia doctor | 12 consultations per individual | € 1,643 | € 1,643 | (2) |
| Consultation with nurse specialist | 12 consultations per individual | € 1,643 | € 1,643 | (2) |
| Consultation with psychologist** | One consultation per individual |  | € 138 | (2) |
| Lab test   - Blood count: Hb, HCT, thrombocyte, leucocyte, differential - Hemostasis: INR, PT, APTT, fibrinogen, d-dimer - Factor IX: one-stage and chromogenic factor activity level, antigen, inhibitor - Liver function: AST, ALT, LDH, ALP, GGT, total bilirubin, albumin | 12 tests per individual | € 3,323 | € 3,323 | (2, 4, 8) |
| Administration of medication for liver function abnormalities^††^   - Corticosteroid: prednisolone 60mg - Extra medication: calcium carbonate with vitamin D, omeprazole, cotrimoxazole - Extra follow-up: glucose, hypertension | 80-day course of corticosteroid treatments per individual | € 159 | € 159 | (4, 7) |
| Consultation with hepatologist^††^ | One consultation per individual | € 137 | € 137 | (2) |
| Manage data register | 12 occurrences per individual | € 1,113 | € 1,113 | ABC for 2 hours per occurrence |
| **Year 1 (Weeks 13-52)** |  | **€ 6,149** | **€ 6,198** |  |
| Consultation with hemophilia doctor | 9 consultations per individual | € 1,232 | € 1,232 | (2) |
| Consultation with nurse specialist | 9 consultations per individual | € 1,232 | € 1,232 | (2) |
| Consultation with psychologist** | One consultation per individual |  | € 138 | (2) |
| Consultation with physical therapist (assessment of joint health)* | Two consultations per individual | € 89 |  | (2) |
| Lab test   - Blood count: Hb, HCT, thrombocyte, leucocyte, differential - Hemostasis: INR, PT, APTT, fibrinogen, d-dimer - Factor IX: one-stage and chromogenic factor activity level, antigen, inhibitor - Liver function: AST, ALT, LDH, ALP, GGT, total bilirubin, albumin | 9 tests per individual | € 2,492 | € 2,492 | (2, 4, 8) |
| Administration of medication for liver function abnormalities   - Corticosteroid: prednisolone 60mg - Extra medication: calcium carbonate with vitamin D, omeprazole, cotrimoxazole - Extra follow-up: glucose, hypertension | Assumed liver function abnormalities resolved within the first 12 weeks |  |  |  |
| Liver ultrasound | Two assessments per individual | € 269 | € 269 | (2) |
| Consultation with hepatologist | Assumed liver function abnormalities resolved within the first 12 weeks |  |  |  |
| Manage data register | 9 occurrences per individual | € 835 | € 835 | ABC for 2 hours per occurrence |
| **Year 2** |  | **€ 1,645** | **€ 1,694** |  |
| Consultation with hemophilia doctor | Two consultations per individual | € 274 | € 274 | (2) |
| Consultation with nurse specialist | Two consultations per individual | € 274 | € 274 | (2) |
| Consultation with psychologist** | One consultation per individual |  | € 138 | (2) |
| Consultation with physical therapist (assessment of joint health)* | Two consultations per individual | € 89 |  | (2) |
| Lab test   - Blood count: Hb, HCT, thrombocyte, leucocyte, differential - Hemostasis: INR, PT, APTT, fibrinogen, d-dimer - Factor IX: one-stage and chromogenic factor activity level, antigen, inhibitor - Liver function: AST, ALT, LDH, ALP, GGT, total bilirubin, albumin | Two tests per individual | € 554 | € 554 | (2, 4, 8) |
| Administration of medication for liver function abnormalities   - Corticosteroid: prednisolone 60mg - Extra medication: calcium carbonate with vitamin D, omeprazole, cotrimoxazole - Extra follow-up: glucose, hypertension | Assumed liver function abnormalities resolved within the first year |  |  |  |
| Liver ultrasound | Two assessments per individual | € 269 | € 269 | (2) |
| Consultation with hepatologist | Assumed liver function abnormalities resolved within the first year |  |  |  |
| Manage data register | Two occurrences per individual | € 186 | € 186 | ABC for 2 hours per occurrence |

*A care activity that was conducted in the clinical trial and will *not* continue in real-world practice. **A care activity that was *not* conducted in the clinical trial but will be provided in real-world practice. ^†^The estimation of follow-up costs encompassed the expenses associated with managing liver function abnormalities. ^††^The costs for administering medication for liver function abnormalities and consultations with a hepatologist were €0 if the eligible individual remained free of liver function abnormalities during the follow-up period. *AAV5* indicates adeno-associated virus 5; *ABC,* activity-based costing; *AFP,* alpha-fetoprotein; *ALP,* alkaline phosphatase; *ALT,* alanine aminotransferase; *APTT,* activated partial thromboplastin time; *AST,* aspartate aminotransferase; *CRP,* C-reactive protein; *DNA,* deoxyribonucleic acid; *ESO,* environmental safety officer; *ESR,* erythrocyte sedimentation rate; *GGT*, gamma-glutamyl transferase; *GMO,* genetically modified organisms; *Hb,* hemoglobin; *HBsAg,* hepatitis B surface antigen; *HBV*, hepatitis B virus; *HCT,* hematocrit; *HCV*, hepatitis C virus; *HIV*, human immunodeficiency virus, *IM-MV,* Environment-Medical Veterinary Research; *INR,* international normalized ratio; *LDH,* lactate dehydrogenase; *NZA,* Nederlandse Zorgautoriteit; *PIVKA-II,* protein induced by vitamin K absence-II, *PT*, prothrombin time; *RNA,* ribonucleic acid; *ZIN,* Zorginstituut Nederland.

**Table 3** Sensitivity analyses of resources used and associated costs for delivering gene therapy for hemophilia A

| **Care activity (hospital perspective)** | **Frequency per unit** | **Clinical trial** | | **Clinical practice** | | **Source of min-max** |
| --- | --- | --- | --- | --- | --- | --- |
|  |  | **Min** | **Max** | **Min** | **Max** |  |
| **Site preparation** |  | **€ 8,407** | **€ 25,295** | **€ 5,305** | **€ 12,889** |  |
| Labor cost of ESO |  | € 3,850 | € 15,400 | € 875 | € 3,500 |  |
| Determine the specific permit required* | One-time charge per center | € 875 | € 3,500 |  |  | (1) |
| Application of IM-MV permit* | One-time charge per center | € 1,050 | € 4,200 |  |  | (1) |
| Maintenance of IM-MV module throughout the trial period* | One-time charge per center | € 1,050 | € 4,200 |  |  | (1) |
| Develop safety manual | One-time charge per center | € 875 | € 3,500 | € 875 | € 3,500 | (1) |
| Labor cost of hospital pharmacist |  | € 253 | € 886 | € 127 | € 380 |  |
| Evaluate study protocol* | One-time charge per center | € 127 | € 506 |  |  | Time variation (1-4 hours) |
| Develop guideline for preparing the GMO | One-time charge per center | € 127 | € 380 | € 127 | € 380 | Time variation (1-3 hours) |
| Train and educate involved personnel |  | € 4,304 | € 9,009 | € 4,304 | € 9,009 |  |
| Handling and preparation of the GMO  (1 hospital pharmacist and 2 pharmacy technicians) | One-time charge per center | € 3,902 | € 7,805 | € 3,902 | € 7,805 | Time variation (24-32 hours) |
| Knowledge about GT and infusion process  (2 hemophilia doctors, 1 nurse specialist, 1 nurse consultant) | One-time charge per center | € 401 | € 1,204 | € 401 | € 1,204 | Time variation (1-3 hours) |
| **Screening** |  | **€ 1,657** | **€ 5,056** | **€ 1,723** | **€ 5,152** |  |
| Consultation with healthcare professionals |  | € 679 | € 1,297 | € 746 | € 1,392 |  |
| Consultation with hemophilia doctor | 2-4 consultations per individual | € 274 | € 548 | € 274 | € 548 | Number of consultations (2-4 times) |
| Consultation with nurse specialist | 2-4 consultations per individual | € 274 | € 548 | € 274 | € 548 | Number of consultations (2-4 times) |
| Consultation with hepatologist | One consultation for every 8 out of 10 individuals | € 88 | € 131 | € 88 | € 131 | Plus or minus 20% of €110 |
| Consultation with psychologist** | One consultation per individual |  |  | € 110 | € 166 | Plus or minus 20% of €138 |
| Consultation with physical therapist (assessment of joint health)* | One consultation per individual | € 44 | € 70 |  |  | (2, 9-12) |
| Complete lab test |  | € 747 | € 3,200 | € 747 | € 3,200 |  |
| AAV5 diagnostic test | One test per individual | € 14 | € 25 | € 14 | € 25 | (3) |
| Blood count: Hb, HCT, thrombocyte, leucocyte, differential | One test per individual | € 9 | € 106 | € 9 | € 106 | (4, 9-12) |
| Hemostasis: INR, PT, APTT, fibrinogen, d-dimer | One test per individual | € 32 | € 54 | € 32 | € 54 | (4, 9-12) |
| Factor VIII activity level (one-stage assay) |  | € 16 | € 93 | € 16 | € 93 | (4, 9-12) |
| Factor VIII activity level (chromogenic assay) | One test per individual | € 53 | € 63 | € 53 | € 63 | (4, 9-12) |
| Factor VIII antigen | One test per individual | € 51 | € 105 | € 51 | € 105 | (4, 9-12) |
| Factor VIII inhibitor | One test per individual | € 8 | € 11 | € 8 | € 11 | Assumed to have an equal cost that of factor IX inhibitor |
| Liver function: AST, ALT, LDH, ALP, GGT, total bilirubin, albumin | Two tests per individual | € 29 | € 491 | € 29 | € 491 | (2, 4, 9-12) |
| Renal function: creatinine, urea | One test per individual | € 2 | € 4 | € 2 | € 4 | (2, 4, 9-12) |
| Virology: hepatitis serology, HBsAg, HBV DNA, anti-HCV, HCV RNA, HIV serology | One test per individual | € 490 | € 1,978 | € 490 | € 1,978 | (4, 9-12) |
| Tumor marker: AFP, PIVKA-II | One test per individual | € 36 | € 256 | € 36 | € 256 | (4, 9-12) |
| Inflammatory marker: CRP, ESR | One test per individual | € 7 | € 13 | € 7 | € 13 | (2, 4, 9-12) |
| Liver function assessment |  | € 230 | € 560 | € 230 | € 560 |  |
| Liver ultrasound | One assessment per individual | € 112 | € 236 | € 112 | € 236 | (2, 4, 9-12) |
| FibroScan | One assessment per individual | € 118 | € 324 | € 118 | € 324 | (2, 4, 9-12) |
| **Pre-treatment preparation** |  | **€ 1,576** | **€ 4,637** | **€ 1,576** | **€ 4,637** |  |
| Labor cost of hospital pharmacist |  | € 158 | € 759 | € 158 | € 759 |  |
| Order process of the GMO | One occurrence per individual | € 32 | € 127 | € 32 | € 127 | Time variation (0.25-1 hour) |
| Receive and quarantine the GMO upon delivery | One occurrence per individual | € 32 | € 127 | € 32 | € 127 | Time variation (0.25-1 hour) |
| Conduct quality inspection and release the GMO | One occurrence per individual | € 32 | € 127 | € 32 | € 127 | Time variation (0.25-1 hour) |
| Complete administrative tasks for pharmacy operations | One occurrence per individual | € 63 | € 380 | € 63 | € 380 | Time variation (0.5-3 hours) |
| Labor cost of nurse specialist |  | € 1,113 | € 2,782 | € 1,113 | € 2,782 |  |
| Manage logistical arrangements for administration day | One occurrence per individual | € 1,113 | € 2,782 | € 1,113 | € 2,782 | Time variation (16-40 hours) |
| Preparation of gene therapy infusion |  | € 305 | €1,097 | € 305 | €1,097 |  |
| *Labor cost of hospital pharmacist* |  | € 42 | €149 | € 42 | €149 |  |
| Confirm the prescription and release the GMO | One occurrence per individual | € 17 | € 50 | € 17 | € 50 | Time variation (0.16-0.5 hour) |
| Confirm guideline adherence and release the infusion bag | One occurrence per individual | € 25 | € 100 | € 25 | € 100 | Time variation (0.25-1 hour) |
| *Labor cost of pharmacy technician* |  | € 92 | € 555 | € 92 | € 555 |  |
| Prepare the cleanroom facility | One occurrence per individual | € 23 | € 185 | € 23 | € 185 | Time variation (0.25-2 hours) |
| Actual preparation work by two pharmacy technicians | One occurrence per individual | € 46 | € 185 | € 46 | € 185 | Time variation (0.5-2 hours) |
| Clean the cleanroom facility after preparation | One occurrence per individual | € 23 | € 185 | € 23 | € 185 | Time variation (0.25-2 hours) |
| *Materials used for preparation* | One set of material per individual | € 106 | € 159 | € 106 | € 159 | Plus or minus 20% of € 133 |
| *Storage and cleanroom facilities for GMO, and GMO waste management* | One occurrence per individual | € 65 | € 233 | € 65 | € 233 | Overhead costs (calculated as 27% of the total preparation cost) |
| **Administration day** |  | **€ 1,550** | **€ 3,882** | **€ 1,550** | **€ 3,882** |  |
| Admission to inpatient facility including nursing care, room cleaning and medical waste disposal | One occurrence per individual | € 359 | € 539 | € 359 | € 539 | Plus or minus 20% of € 449 |
| Consultation with hemophilia doctor | One consultation per individual | € 137 | € 411 | € 137 | € 411 | Time variation (0.3-1 hour) |
| Infusion of gene therapy |  | € 231 | € 459 | € 231 | € 459 |  |
| Intravenous administration | One occurrence per individual | € 156 | € 234 | € 156 | € 234 | Plus or minus 20% of € 195 |
| Presence of hemophilia doctor during the infusion | One occurrence per individual | € 75 | € 225 | € 75 | € 225 | Time variation (0.5-1.5 hours) |
| Labor cost for post-treatment follow-up  (1 hemophilia doctor and 1 nurse specialist) | One occurrence per individual | € 820 | € 2,460 | € 820 | € 2,460 | Time variation (4-12 hours) |
| Drug costs |  | € 3 | € 13 | € 3 | € 13 |  |
| Administration of medication for allergic reaction (adrenaline) | One administration for every 1-5 out of 10 individuals | € 3 | € 13 | € 3 | € 13 | Proportion of individuals receiving this drug (0.1-0.5) |
| **Follow-up Year 1**^†^ |  | **€ 16,504** | **€ 36,029** | **€ 16,636** | **€ 36,220** |  |
| **Year 1 (Weeks 1-26)** |  | **€ 11,690** | **€ 20,946** | **€ 11,756** | **€ 21,041** |  |
| Consultation with hemophilia doctor | 20-36 consultations per individual | € 2,738 | € 4,928 | € 2,738 | € 4,928 | Number of consultations (20-36 times) |
| Consultation with nurse specialist | 20-36 consultations per individual | € 2,738 | € 4,928 | € 2,738 | € 4,928 | Number of consultations (20-36 times) |
| Consultation with psychologist** | One consultation per individual |  |  | € 110 | € 166 | Plus or minus 20% of € 138 |
| Consultation with physical therapist (assessment of joint health)* | One consultation per individual | € 44 | € 70 |  |  | (2, 9-12) |
| Lab test   - Blood count: Hb, HCT, thrombocyte, leucocyte, differential - Hemostasis: INR, PT, APTT, fibrinogen, d-dimer - Factor VIII: one-stage and chromogenic factor activity level, antigen, inhibitor - Liver function: AST, ALT, LDH, ALP, GGT, total bilirubin, albumin | 20-36 tests per individual | € 3,799 | € 6,837 | € 3,799 | € 6,837 | Number of tests (20-36 times) |
| Administration of medication for liver function abnormalities^††^   - Corticosteroid: prednisolone 60mg - Extra medication: calcium carbonate with vitamin D, omeprazole, cotrimoxazole - Extra follow-up: glucose, hypertension | 183-day course of corticosteroid treatments per individual | € 295 | € 443 | € 295 | € 443 | Plus or minus 20% of € 369 |
| Liver ultrasound | One assessment per individual | € 112 | € 236 | € 112 | € 236 | (2, 4, 9-12) |
| Consultation with hepatologist^††^ | One consultation per individual | € 110 | € 164 | € 110 | € 164 | Plus or minus 20% of € 137 |
| Manage data register | 20-36 occurrences per individual | € 1,855 | € 3,339 | € 1,855 | € 3,339 | Number of occurrences (20-36 times) |
| **Year 1 (Weeks 27-52)** |  | **€ 4,814** | **€ 15,083** | **€ 4,880** | **€ 15,179** |  |
| Consultation with hemophilia doctor | 8-26 consultations per individual | € 1,095 | € 3,559 | € 1,095 | € 3,559 | Number of consultations (8-26 times) |
| Consultation with nurse specialist | 8-26 consultations per individual | € 1,095 | € 3,559 | € 1,095 | € 3,559 | Number of consultations (8-26 times) |
| Consultation with psychologist** | One consultation per individual |  |  | € 110 | € 166 | Plus or minus 20% of € 138 |
| Consultation with physical therapist (assessment of joint health)* | One consultation per individual | € 44 | € 70 |  |  | (2, 9-12) |
| Lab test   - Blood count: Hb, HCT, thrombocyte, leucocyte, differential - Hemostasis: INR, PT, APTT, fibrinogen, d-dimer - Factor VIII: one-stage and chromogenic factor activity level, antigen, inhibitor - Liver function: AST, ALT, LDH, ALP, GGT, total bilirubin, albumin | 8-26 tests per individual | € 1,519 | € 4,938 | € 1,519 | € 4,938 | Number of tests (8-26 times) |
| Administration of medication for liver function abnormalities^††^   - Corticosteroid: prednisolone 60mg - Extra medication: calcium carbonate with vitamin D, omeprazole, cotrimoxazole - Extra follow-up: glucose, hypertension | 60-day course of corticosteroid treatments per individual | € 97 | € 145 | € 97 | € 145 | Plus or minus 20% of € 121 |
| Liver ultrasound | One assessment per individual | € 112 | € 236 | € 112 | € 236 | (2, 4, 9-12) |
| Consultation with hepatologist^††^ | One consultation per individual | € 110 | € 164 | € 110 | € 164 | Plus or minus 20% of € 137 |
| Manage data register | 8-26 occurrences per individual | € 742 | € 2,412 | € 742 | € 2,412 | Number of occurrences (8-26 times) |
| **Year 2** |  | **€ 1,425** | **€ 2,838** | **€ 1,447** | **€ 2,863** |  |
| Consultation with hemophilia doctor | 2-4 consultations per individual | € 274 | € 548 | € 274 | € 548 | Number of consultations (2-4 times) |
| Consultation with nurse specialist | 2-4 consultations per individual | € 274 | € 548 | € 274 | € 548 | Number of consultations (2-4 times) |
| Consultation with psychologist** | One consultation per individual |  |  | € 110 | € 166 | Plus or minus 20% of € 138 |
| Consultation with physical therapist (assessment of joint health)* | Two consultations per individual | € 89 | € 141 |  |  | (2, 9-12) |
| Lab test   - Blood count: Hb, HCT, thrombocyte, leucocyte, differential - Hemostasis: INR, PT, APTT, fibrinogen, d-dimer - Factor VIII: one-stage and chromogenic factor activity level, antigen, inhibitor - Liver function: AST, ALT, LDH, ALP, GGT, total bilirubin, albumin | 2-4 tests per individual | € 380 | € 760 | € 380 | € 760 | Number of tests (2-4 times) |
| Administration of medication for liver function abnormalities   - Corticosteroid: prednisolone 60mg - Extra medication: calcium carbonate with vitamin D, omeprazole, cotrimoxazole - Extra follow-up: glucose, hypertension | Assumed liver function abnormalities resolved within the first year |  |  |  |  |  |
| Liver ultrasound | Two assessments per individual | € 224 | € 471 | € 224 | € 471 | (2, 4, 9-12) |
| Consultation with hepatologist | Assumed liver function abnormalities resolved within the first year |  |  |  |  |  |
| Manage data register | 2-4 occurrences per individual | € 186 | € 371 | € 186 | € 371 | Number of occurrences (2-4 times) |

*A care activity that was conducted in the clinical trial and will *not* continue in real-world practice. **A care activity that was *not* conducted in the clinical trial but will be provided in real-world practice. ^†^The estimation of follow-up costs encompassed the expenses associated with managing liver function abnormalities. ^††^The costs for administering medication for liver function abnormalities and consultations with a hepatologist were €0 if the eligible individual remained free of liver function abnormalities during the follow-up period. *AAV5* indicates adeno-associated virus 5; *ABC,* activity-based costing; *AFP,* alpha-fetoprotein; *ALP,* alkaline phosphatase; *ALT,* alanine aminotransferase; *APTT,* activated partial thromboplastin time; *AST,* aspartate aminotransferase; *CRP,* C-reactive protein; *DNA,* deoxyribonucleic acid; *ESO,* environmental safety officer; *ESR,* erythrocyte sedimentation rate; *GGT*, gamma-glutamyl transferase; *GMO,* genetically modified organisms; *Hb,* hemoglobin*; HBsAg,* hepatitis B surface antigen; *HBV*, hepatitis B virus; *HCT,* hematocrit; *HCV*, hepatitis C virus; *HIV*, human immunodeficiency virus, *IM-MV,* Environment-Medical Veterinary Research; *INR,* international normalized ratio; *LDH,* lactate dehydrogenase; *NZA,* Nederlandse Zorgautoriteit; *PIVKA-II,* protein induced by vitamin K absence-II, *PT*, prothrombin time; *RNA,* ribonucleic acid; *ZIN,* Zorginstituut Nederland.

**Table 4** Sensitivity analyses of resources used and associated costs for delivering gene therapy for hemophilia B

| **Care activity (hospital perspective)** | **Frequency per unit** | **Clinical trial** | | **Clinical practice** | | **Source of min-max** |
| --- | --- | --- | --- | --- | --- | --- |
|  |  | **Min** | **Max** | **Min** | **Max** |  |
| **Site preparation** |  | **€ 8,407** | **€ 25,295** | **€ 5,305** | **€ 12,889** |  |
| Labor cost of ESO |  | € 3,850 | € 15,400 | € 875 | € 3,500 |  |
| Determine the specific permit required* | One-time charge per center | € 875 | € 3,500 |  |  | (1) |
| Application of IM-MV permit* | One-time charge per center | € 1,050 | € 4,200 |  |  | (1) |
| Maintenance of IM-MV module throughout the trial period* | One-time charge per center | € 1,050 | € 4,200 |  |  | (1) |
| Develop safety manual | One-time charge per center | € 875 | € 3,500 | € 875 | € 3,500 | (1) |
| Labor cost of hospital pharmacist |  | € 253 | € 886 | € 127 | € 380 |  |
| Evaluate study protocol* | One-time charge per center | € 127 | € 506 |  |  | Time variation (1-4 hours) |
| Develop guideline for preparing the GMO | One-time charge per center | € 127 | € 380 | € 127 | € 380 | Time variation (1-3 hours) |
| Train and educate involved personnel |  | € 4,304 | € 9,009 | € 4,304 | € 9,009 |  |
| Handling and preparation of the GMO  (1 hospital pharmacist and 2 pharmacy technicians) | One-time charge per center | € 3,902 | € 7,805 | € 3,902 | € 7,805 | Time variation (24-32 hours) |
| Knowledge about GT and infusion process  (2 hemophilia doctors, 1 nurse specialist, 1 nurse consultant) | One-time charge per center | € 401 | € 1,204 | € 401 | € 1,204 | Time variation (1-3 hours) |
| **Screening** |  | **€ 1,670** | **€ 5,016** | **€ 1,736** | **€ 5,111** |  |
| Consultation with healthcare professionals |  | € 614 | € 1,198 | € 680 | € 1,294 |  |
| Consultation with hemophilia doctor | 2-4 consultations per individual | € 274 | € 548 | € 274 | € 548 | Number of consultations (2-4 times) |
| Consultation with nurse specialist | 2-4 consultations per individual | € 274 | € 548 | € 274 | € 548 | Number of consultations (2-4 times) |
| Consultation with hepatologist | One consultation for every 2 out of 10 individuals | € 22 | € 33 | € 22 | € 33 | Plus or minus 20% of €27 |
| Consultation with psychologist** | One consultation per individual |  |  | € 110 | € 166 | Plus or minus 20% of €138 |
| Consultation with physical therapist (assessment of joint health)* | One consultation per individual | € 44 | € 70 |  |  | (2, 9-12) |
| Complete lab test |  | € 827 | € 3,258 | € 827 | € 3,258 |  |
| AAV5 diagnostic test | One test per individual | € 14 | € 25 | € 14 | € 25 | (3) |
| Blood count: Hb, HCT, thrombocyte, leucocyte, differential | One test per individual | € 9 | € 106 | € 9 | € 106 | (4, 9-12) |
| Hemostasis: INR, PT, APTT, fibrinogen, d-dimer | One test per individual | € 32 | € 54 | € 32 | € 54 | (4, 9-12) |
| Factor IX activity level (one-stage assay) | One test per individual | € 37 | € 54 | € 37 | € 54 | (4, 9-12) |
| Factor IX activity level (chromogenic assay) | One test per individual | € 112 | € 161 | € 112 | € 161 | Assumed to have a cost three times that of factor IX activity level one-stage assay |
| Factor IX antigen | One test per individual | € 51 | € 105 | € 51 | € 105 | Assumed to have an equal cost that of factor VIII antigen |
| Factor IX inhibitor | One test per individual | € 8 | € 11 | € 8 | € 11 | Plus or minus 20% of €9 |
| Liver function: AST, ALT, LDH, ALP, GGT, total bilirubin, albumin | Two tests per individual | € 29 | € 491 | € 29 | € 491 | (2, 4, 9-12) |
| Renal function: creatinine, urea | One test per individual | € 2 | € 4 | € 2 | € 4 | (2, 4, 9-12) |
| Virology: hepatitis serology, HBsAg, HBV DNA, anti-HCV, HCV RNA, HIV serology | One test per individual | € 490 | € 1,978 | € 490 | € 1,978 | (4, 9-12) |
| Tumor marker: AFP, PIVKA-II | One test per individual | € 36 | € 256 | € 36 | € 256 | (4, 9-12) |
| Inflammatory marker: CRP, ESR | One test per individual | € 7 | € 13 | € 7 | € 13 | (2, 4, 9-12) |
| Liver function assessment |  | € 230 | € 560 | € 230 | € 560 |  |
| Liver ultrasound | One assessment per individual | € 112 | € 236 | € 112 | € 236 | (2, 4, 9-12) |
| FibroScan | One assessment per individual | € 118 | € 324 | € 118 | € 324 | (2, 4, 9-12) |
| **Pre-treatment preparation** |  | **€ 1,576** | **€ 4,637** | **€ 1,576** | **€ 4,637** |  |
| Labor cost of hospital pharmacist |  | € 158 | € 759 | € 158 | € 759 |  |
| Order process of the GMO | One occurrence per individual | € 32 | € 127 | € 32 | € 127 | Time variation (0.25-1 hour) |
| Receive and quarantine the GMO upon delivery | One occurrence per individual | € 32 | € 127 | € 32 | € 127 | Time variation (0.25-1 hour) |
| Conduct quality inspection and release the GMO | One occurrence per individual | € 32 | € 127 | € 32 | € 127 | Time variation (0.25-1 hour) |
| Complete administrative tasks for pharmacy operations | One occurrence per individual | € 63 | € 380 | € 63 | € 380 | Time variation (0.5-3 hours) |
| Labor cost of nurse specialist |  | € 1,113 | € 2,782 | € 1,113 | € 2,782 |  |
| Manage logistical arrangements for administration day | One occurrence per individual | € 1,113 | € 2,782 | € 1,113 | € 2,782 | Time variation (16-40 hours) |
| Preparation of gene therapy infusion |  | € 305 | €1,097 | € 305 | €1,097 |  |
| *Labor cost of hospital pharmacist* |  | € 42 | €149 | € 42 | €149 |  |
| Confirm the prescription and release the GMO | One occurrence per individual | € 17 | € 50 | € 17 | € 50 | Time variation (0.16-0.5 hour) |
| Confirm guideline adherence and release the infusion bag | One occurrence per individual | € 25 | € 100 | € 25 | € 100 | Time variation (0.25-1 hour) |
| *Labor cost of pharmacy technician* |  | € 92 | € 555 | € 92 | € 555 |  |
| Prepare the cleanroom facility | One occurrence per individual | € 23 | € 185 | € 23 | € 185 | Time variation (0.25-2 hours) |
| Actual preparation work by two pharmacy technicians | One occurrence per individual | € 46 | € 185 | € 46 | € 185 | Time variation (0.5-2 hours) |
| Clean the cleanroom facility after preparation | One occurrence per individual | € 23 | € 185 | € 23 | € 185 | Time variation (0.25-2 hours) |
| *Materials used for preparation* | One set of material per individual | € 106 | € 159 | € 106 | € 159 | Plus or minus 20% of € 133 |
| *Storage and cleanroom facilities for GMO, and GMO waste management* | One occurrence per individual | € 65 | € 233 | € 65 | € 233 | Overhead costs (calculated as 27% of the total preparation cost) |
| **Administration day** |  | **€ 1,550** | **€ 3,882** | **€ 1,550** | **€ 3,882** |  |
| Admission to inpatient facility including nursing care, room cleaning and medical waste disposal | One occurrence per individual | € 359 | € 539 | € 359 | € 539 | Plus or minus 20% of € 449 |
| Consultation with hemophilia doctor | One consultation per individual | € 137 | € 411 | € 137 | € 411 | Time variation (0.3-1 hour) |
| Infusion of gene therapy |  | € 231 | € 459 | € 231 | € 459 |  |
| Intravenous administration | One occurrence per individual | € 156 | € 234 | € 156 | € 234 | Plus or minus 20% of € 195 |
| Presence of hemophilia doctor during the infusion | One occurrence per individual | € 75 | € 225 | € 75 | € 225 | Time variation (0.5-1.5 hours) |
| Labor cost for post-treatment follow-up  (1 hemophilia doctor and 1 nurse specialist) | One occurrence per individual | € 820 | € 2,460 | € 820 | € 2,460 | Time variation (4-12 hours) |
| Drug costs |  | € 3 | € 13 | € 3 | € 13 |  |
| Administration of medication for allergic reaction (adrenaline) | One administration for every 1-5 out of 10 individuals | € 3 | € 13 | € 3 | € 13 | Proportion of individuals receiving this drug (0.1-0.5) |
| **Follow-up Year 1**^†^ |  | **€ 13,418** | **€ 22,844** | **€ 13,550** | **€ 23,034** |  |
| **Year 1 (Weeks 1-12)** |  | **€ 7,958** | **€ 15,797** | **€ 8,068** | **€ 15,963** |  |
| Consultation with hemophilia doctor | 12-24 consultations per individual | € 1,643 | € 3,285 | € 1,643 | € 3,285 | Number of consultations (12-24 times) |
| Consultation with nurse specialist | 12-24 consultations per individual | € 1,643 | € 3,285 | € 1,643 | € 3,285 | Number of consultations (12-24 times) |
| Consultation with psychologist** | One consultation per individual |  |  | € 110 | € 166 | Plus or minus 20% of € 138 |
| Lab test   - Blood count: Hb, HCT, thrombocyte, leucocyte, differential - Hemostasis: INR, PT, APTT, fibrinogen, d-dimer - Factor VIII: one-stage and chromogenic factor activity level, antigen, inhibitor - Liver function: AST, ALT, LDH, ALP, GGT, total bilirubin, albumin | 12-24 tests per individual | € 3,323 | € 6,646 | € 3,323 | € 6,646 | Number of tests (12-24 times) |
| Administration of medication for liver function abnormalities^††^   - Corticosteroid: prednisolone 60mg - Extra medication: calcium carbonate with vitamin D, omeprazole, cotrimoxazole - Extra follow-up: glucose, hypertension | 80-day course of corticosteroid treatments per individual | € 127 | € 190 | € 127 | € 190 | Plus or minus 20% of € 159 |
| Consultation with hepatologist^††^ | One consultation per individual | € 110 | € 164 | € 110 | € 164 | Plus or minus 20% of € 137 |
| Manage data register | 12-24 occurrences per individual | € 1,113 | € 2,226 | € 1,113 | € 2,226 | Number of occurrences (12-24 times) |
| **Year 1 (Weeks 13-52)** |  | **€ 5,460** | **€ 7,046** | **€ 5,482** | **€ 7,071** |  |
| Consultation with hemophilia doctor | 8-10 consultations per individual | € 1,095 | € 1,369 | € 1,095 | € 1,369 | Number of consultations (8-10 times) |
| Consultation with nurse specialist | 8-10 consultations per individual | € 1,095 | € 1,369 | € 1,095 | € 1,369 | Number of consultations (8-10 times) |
| Consultation with psychologist** | One consultation per individual |  |  | € 110 | € 166 | Plus or minus 20% of € 138 |
| Consultation with physical therapist (assessment of joint health)* | Two consultations per individual | € 89 | € 141 |  |  | (2, 9-12) |
| Lab test   - Blood count: Hb, HCT, thrombocyte, leucocyte, differential - Hemostasis: INR, PT, APTT, fibrinogen, d-dimer - Factor VIII: one-stage and chromogenic factor activity level, antigen, inhibitor - Liver function: AST, ALT, LDH, ALP, GGT, total bilirubin, albumin | 8-10 tests per individual | € 2,215 | € 2,769 | € 2,215 | € 2,769 | Number of tests (8-10 times) |
| Administration of medication for liver function abnormalities   - Corticosteroid: prednisolone 60mg - Extra medication: calcium carbonate with vitamin D, omeprazole, cotrimoxazole - Extra follow-up: glucose, hypertension | Assumed liver function abnormalities resolved within the first 12 weeks |  |  |  |  |  |
| Liver ultrasound | Two assessments per individual | € 224 | € 471 | € 224 | € 471 | (2, 4, 9-12) |
| Consultation with hepatologist | Assumed liver function abnormalities resolved within the first 12 weeks |  |  |  |  |  |
| Manage data register | 8-10 occurrences per individual | € 742 | € 928 | € 742 | € 928 | Number of occurrences (8-10 times) |
| **Year 2** |  | **€ 956** | **€ 2,542** | **€ 977** | **€ 2,567** |  |
| Consultation with hemophilia doctor | 1-3 consultations per individual | € 137 | € 411 | € 137 | € 411 | Number of consultations (1-3 times) |
| Consultation with nurse specialist | 1-3 consultations per individual | € 137 | € 411 | € 137 | € 411 | Number of consultations (1-3 times) |
| Consultation with psychologist** | One consultation per individual |  |  | € 110 | € 166 | Plus or minus 20% of € 138 |
| Consultation with physical therapist (assessment of joint health)* | Two consultations per individual | € 89 | € 141 |  |  | (2, 9-12) |
| Lab test   - Blood count: Hb, HCT, thrombocyte, leucocyte, differential - Hemostasis: INR, PT, APTT, fibrinogen, d-dimer - Factor VIII: one-stage and chromogenic factor activity level, antigen, inhibitor - Liver function: AST, ALT, LDH, ALP, GGT, total bilirubin, albumin | 1-3 tests per individual | € 277 | € 831 | € 277 | € 831 | Number of tests (1-3 times) |
| Administration of medication for liver function abnormalities   - Corticosteroid: prednisolone 60mg - Extra medication: calcium carbonate with vitamin D, omeprazole, cotrimoxazole - Extra follow-up: glucose, hypertension | Assumed liver function abnormalities resolved within the first year |  |  |  |  |  |
| Liver ultrasound | Two assessments per individual | € 224 | € 471 | € 224 | € 471 | (2, 4, 9-12) |
| Consultation with hepatologist | Assumed liver function abnormalities resolved within the first year |  |  |  |  |  |
| Manage data register | 1-3 occurrences per individual | € 93 | € 278 | € 93 | € 278 | Number of occurrences (1-3 times) |

*A care activity that was conducted in the clinical trial and will *not* continue in real-world practice. **A care activity that was *not* conducted in the clinical trial but will be provided in real-world practice. ^†^The estimation of follow-up costs encompassed the expenses associated with managing liver function abnormalities.

^††^The costs for administering medication for liver function abnormalities and consultations with a hepatologist were €0 if the eligible individual remained free of liver function abnormalities during the follow-up period. *AAV5* indicates adeno-associated virus 5; *ABC,* activity-based costing; *AFP,* alpha-fetoprotein; *ALP,* alkaline phosphatase; *ALT,* alanine aminotransferase; *APTT,* activated partial thromboplastin time; *AST,* aspartate aminotransferase; *CRP,* C-reactive protein; *DNA,* deoxyribonucleic acid; *ESO,* environmental safety officer; *ESR,* erythrocyte sedimentation rate; *GGT*, gamma-glutamyl transferase; *GMO,* genetically modified organisms; *Hb,* hemoglobin; *HBsAg,* hepatitis B surface antigen; *HBV*, hepatitis B virus; *HCT,* hematocrit; *HCV*, hepatitis C virus; *HIV*, human immunodeficiency virus, *IM-MV,* Environment-Medical Veterinary Research; *INR,* international normalized ratio; *LDH,* lactate dehydrogenase; *NZA,* Nederlandse Zorgautoriteit; *PIVKA-II,* protein induced by vitamin K absence-II, *PT*, prothrombin time; *RNA,* ribonucleic acid; *ZIN,* Zorginstituut Nederland.

**References**

1. Kempen KEB-v, Overbeeke JM. Gene Therapy, Environmental Safety Officer Rate. [in Dutch]. Internal report. 2024.

2. Roijen LHv, Peeters S, Kanters T. Costing manual: Methods and Reference Prices for Economic Evaluations in Healthcare. 2024.

3. Cook K, Forbes SP, Adamski K, Ma JJ, Chawla A, Garrison LP, Jr. Assessing the potential cost-effectiveness of a gene therapy for the treatment of hemophilia A. J Med Econ. 2020;23(5):501-12.

4. DBC product-finder for tariffs.: Dutch Healthcare Authority (Nederlandse Zorgautoriteit; NZa); 2024 [Available from: <https://zorgproducten.nza.nl>.

5. Franken MG, Kanters TA, Coenen JL, de Jong P, Koene HR, Lugtenburg PJ, et al. Potential cost savings owing to the route of administration of oncology drugs: a microcosting study of intravenous and subcutaneous administration of trastuzumab and rituximab in the Netherlands. Anticancer Drugs. 2018;29(8):791-801.

6. Mihajlovic J, Bax P, van Breugel E, Blommestein HM, Hoogendoorn M, Hospes W, et al. Microcosting Study of Rituximab Subcutaneous Injection Versus Intravenous Infusion. Clin Ther. 2017;39(6):1221-32 e4.

7. Drug costs in the Netherlands. [in Dutch]: Dutch National Health Institute (Zorginstituut Nederland; ZIN); 2024 [Available from: <https://www.medicijnkosten.nl>.

8. Bolous N, Chen Y, Wang H, Davidoff AM. The cost-effectiveness of gene therapy for severe hemophilia B: a microsimulation study from the United States perspective. Blood. 2021;138(18):1677-90.

9. Prices for patients with uncontracted care Amsterdam UMC. [in Dutch]. 2024.

10. Prices for patients with uncontracted care Erasmus MC. [in Dutch]. 2024.

11. Prices for patients with uncontracted care UMCU. [in Dutch]. 2024.

12. Prices for patients with uncontracted care UMCG. [in Dutch]. 2024.
